# Innate [^18^F]Fluorodeoxyglucose PET bone networks of lung cancer patients predict survival

**DOI:** 10.1007/s00259-025-07388-8

**Published:** 2025-06-05

**Authors:** Rucha Ronghe, Teresa Crespo Gonzalez, Catriona Wimberley, Karla Suchacki, Adriana A. S. Tavares

**Affiliations:** 1 Edinburgh Medical School, University of Edinburgh, 49 Little France Crescent, Edinburgh, EH16 4SB UK; 2 Edinburgh Medical School: Biomedical Sciences, University of Edinburgh, Old Medical School (Doorway 3), Teviot Place, Edinburgh, EH8 9AG UK; 3 British Heart Foundation-University of Edinburgh Centre for Cardiovascular Science, University of Edinburgh, 47 Little France Crescent, Edinburgh, EH16 4TJ UK; 4 Edinburgh Imaging, University of Edinburgh, Edinburgh, EH16 4TJ UK; 5 School of Physics and Astronomy, University of Edinburgh, Edinburgh, EH9 3JW UK; 6 SRUC, The Roslin Institute, Edinburgh, UK

**Keywords:** Lung cancer, PET/CT, [^18^F]FDG, Network analysis, Survival

## Abstract

**Purpose:**

Prognostication of lung cancer patients remains challenging clinically. This study aims to address this problem by investigating the utility of bone glucose metabolism networks as a prognostic biomarker of lung cancer patients. The rationale for targeting bone metabolic networks specifically comes from the long-recognised role of bone in innate immunity and the recent appreciation of the key role bones play in regulating whole-body glucose metabolism.

**Methods:**

This study is a retrospective analysis of data from the multi-centre trial ACRIN 6668 on non-small cell lung carcinoma (NSCLC). Conventional [^18^F]Fluorodeoxyglucose (FDG) Positron Emission Tomography (PET) standardised uptake value (SUV) analysis of lung lesions and bones as well as network analysis of bones were carried out in 34 stage IIIB non-operable NSCLC patients before and after chemoradiation.

**Results:**

Conventional lung tumour SUV peak analysis of PET data cannot predict NSCLC patient survival (*p* = 0.23–0.35), while network analysis of bone glucose metabolism pre-chemoradiation can significantly predict patient survival (*p* = 0.0003) and identify three distinct clusters of survivors (short-term, long-term and mix-term). Chemoradiation treatment results in homogenisation of the innate survivors’ clusters into adapted mix-term clusters and loss of predictive value of bone glucose metabolism networks (*p* = 0.77).

**Conclusions:**

Innate pre-treatment skeletal glucose metabolism networks of NSCLC patients can predict patient survival in contrast with adapted post-treatment networks, supporting the role of bones in both innate immunity and regulation of systemic glucose metabolism. Newly proposed network analysis performs better than conventional SUV PET analysis, and should be considered as a prognostication tool in the management of NSCLC patients.

**Supplementary Information:**

The online version contains supplementary material available at 10.1007/s00259-025-07388-8.

## Introduction

Cancer is one of the leading causes of death worldwide [1]. Among all types of cancer, lung cancer remains a major killer worldwide [1, 2]. Non-small cell lung carcinoma (NSCLC) is the most common subtype of lung cancer [3]. [^18^F]Fluorodeoxyglucose (FDG) Positron Emission Tomography (PET) is used to assess the malignancy of lung tumours and identify mediastinal lymph node and distant metastases, helping in diagnosis and staging of NSCLC patients [4–9]. [^18^F]FDG PET is also used to assess lung cancer treatment response clinically [10], but its utility as a prognostic biomarker remains challenging [11–13]. [^18^F]FDG has been used as a marker of glucose metabolism in vivo and non-invasively for over 40 years. Activated immune cells have a higher glucose demand thus [^18^F]FDG has been used as a marker of immune responses throughout the body [14].

Changes in whole-body multi-organ glucose metabolism of lung cancer patients versus healthy individuals have been reported [12]. It is now known that bones act as key regulators of whole-body glucose metabolism and studies have elucidated the many novel pathways and mechanisms by which bones achieve this [15–18]. The role of bones and bone cells in innate immunity responses has long been recognised, a concept referred to as osteoimmunity [19, 20]. For example, osteoclasts are derived from immune cells and are important regulators of innate immunity [20, 21]. Evidence has also emerged on how osteoblasts remotely supply lung tumours with cancer-promoting neutrophils in a mouse model [22]. Previously, we have shown that skeletal glucose metabolism forms a complex physiological network in the mouse [23], which could not be identified by conventional [^18^F]FDG PET analysis used routinely in clinical practice.

This study aims to test the hypothesis that if skeletal energy networks are analysed in NSCLC patients, then they can serve as prognostic markers, given the role of bones in both innate immunity and regulation of systemic glucose metabolism. Furthermore, it aims to investigate how bone glucose metabolic networks compare to commonly used clinical measurements in predicting cancer patients’ outcomes before and after treatment.

## Methods

### Data acquisition and study sample

Data were acquired from the ACRIN 6668 study on NSCLC through The Cancer Imaging Archive (TCIA) [24, 25] clinical trial number NCT00083083. The ACRIN 6668 study consisted of imaging participants with inoperable stage IIB/IIIA/IIIB NSCLC, determined using the 1997 AJCC staging criteria, scheduled to be treated with conventional chemoradiation. Participants had to be over 18 and have a blood glucose of ≤ 200 mg/dL. The original study aim was to determine the extent to which SUV before and after treatment could be used to predict clinical outcomes. This clinical trial was conducted in accordance with the Declaration of Helsinki.

The clinical data for the ACRIN 6668 study were extracted from TCIA as two sets of XLS files and subsequently compiled into a summary file for use in the current study. Of the 250 patients registered, 34 were selected for this study based on being all at the same disease stage IIIB and the absence of bone metastases, which was excluded through PET/CT review by a radiologist. All patients had available pre- and post-treatment whole-body PET/CT images, complete clinical data (including systemic glucose values, date of last clinical assessment, and date of last contact or death), successful radiotracer injection without infiltration, and no administration of oral or intravenous contrast. Of the 34 patients, 47.06% (*n* = 16) were female and 52.94% (*n* = 18) male. The mean age was 60.68 years and the mean life expectancy 636.53 days. The pre-treatment mean weight and body mass index (BMI) were 71.75 kg and 25.3 kg/m^2^, respectively, and the mean blood glucose 97.33 mg/dL. The post-treatment mean weight and BMI was 69.46 kg and 24.5 kg/m^2^ respectively, and the mean blood glucose 100.28 mg/dL (Table 1).

Participants were imaged using an ACRIN-approved PET/CT scanner before treatment and relevant clinical data, such as sex, age, weight, and height were collected. Participants fasted for 4 h prior to the [^18^F]FDG injection, 5.18–7.77 MBq/kg, blood glucose was measured two hours before the scan and imaging started 50–70 min after the [^18^F]FDG injection. Participants then underwent 60 + Gy thoracic radiotherapy and concurrent doublet chemotherapy, including a platinum-based agent and a non-platinum, non-gemcitabine agent. Participants were imaged again using the same ACRIN-approved scanner, and methodology, 14 weeks (± 2 weeks) after treatment completion. All institutions had Institutional Review Board (IRB) approval and all participants provided study-specific informed consent. All data collection and management were conducted by the Biostatistics and Data Management Centre at ACRIN, using a built-in security feature that encrypts data. PET images were reconstructed using OSEM with 8 subsets and 2, 3, or 4 iterations and smoothing of 6-mm 3D Gaussian kernel [25].

**Table 1:** Patient demographics and chemotherapy treatment summary table

	Total number (*n* = 34)	(%)
Sex:		
Male	18	52.9
Female	16	47.1
Disease Stage	IIIB (no bone metastasis)	
Mean age (years):	60.68 ± 9.86	
Males	59.78 ± 10.21	
Females	61.69 ± 10.21	
Chemotherapy agents:		
Platinum based		
Cisplatin	15	44.1
Carboplatin	18	52.9
None	1	2.9
Non-platinum based		
Etoposide	8	23.5
Paclitaxel	13	38.2
Taxotere	4	11.8
Irinotecan	5	14.7
Navelbine	4	11.8

### Image analysis

Image analysis of the PET and CT images used in this study was conducted using PMOD 4.0 software (PMOD Technologies, Switzerland). The CT image was segmented using a HU value of > 300, isolating the bones from the rest of the body, following a previously published protocol [26]. A whole-body volume of interest (VOI) was created, outlining the skeleton. VOIs for individual bones (skull, sternum, right and left humerus, right and left femur, spine C1 to L5 and spine L2 to L4) were manually outlined and then intersected with the whole-body skeletal VOI, creating individual VOIs for each bone including skeletal tissue but not bone marrow. Metastases in lung cancer patients have the greatest prevalence in both the thoracic and lumbar regions of the spine [27, 28], however, lumbar metastasis lesions are associated with a mean survival time that is half of that associated with thoracic lesions [28]. Therefore, this study specifically focused on lumbar spine yet lesion free analysis. L2 to L4 vertebrae were prioritised in this network analysis study focusing on the prognostication of NSCLC patients [27, 28]. Due to the small cohort size, the selection of L2 to L4 improves the power of this study, but it is important to highlight that this has the potential to introduce bias.

PET images were first transformed using the body weight SUV image analysis function of PMOD, correcting for time delays, and filling in patient weight and injected dose. Average SUV values for each bone were extracted using the outlined VOIs. Average bone SUVs were then compiled into an Excel file, the SUV humerus value was taken from the arm that was not injected with [^18^F]FDG. The SUV femur value was the average of the left and right femoral values since research shows no variation between the SUV of left and right long bones in the lower extremities [29]. Lung SUV peak values were obtained directly from the ACRIN 6668 data files from previously published analysis [11] and used for group comparisons as well as survival plot analysis.

### Network analysis

Network analysis was conducted using the open-source Graphia 3.0 software (Graphia Technologies, UK) [30]. The Graphia visual analytics platform allows the study of interactions, providing a navigable environment to explore and interpret large and complex topologies. Graphia uses the k-NN algorithm [31] for edge pruning, and the MCL [32] graph clustering algorithm.

First, SUV values and relevant clinical data (age, sex, weight, blood glucose, lung SUV peak, and life expectancy) were summarized into.csv files and then uploaded onto Graphia. The six individual bone SUV values for each patient were combined to form a metabolic profile, shown as a curve, each data point in that curve representing one bone. A relationship graph is then created using these metabolic profiles where each node represents one patient. The pre- and post-treatment PET graphs used a minimum Pearson’s correlation coefficient between nodes of 0.72, where all the edges between nodes show a correlation of ≥ 0.72, with a k value of 5, and Markoff Clustering (MCL) algorithm with a granularity of 2.00. Pearson’s correlation coefficients were adjusted to 0.72 to establish as strong a correlation as possible between datapoints (i.e. patients), while ensuring none were excluded from the networks. We have successfully used similar Pearson’s correlation values (> 0.6) to robustly characterise bone networks from mice using varying datasets with different n numbers, different scanning length and different noise levels [23, 33]. MCL creates clusters, or groups of nodes, based on how closely correlated they are, where granularity refers to the sensitivity of the algorithm and a value of 2 was found to provide stable cluster numbers. It is known that data distributions often exhibit multiple granularities, which once optimised can enhance k-NN classification and clustering methods [34]. Graphia only allows for single granularity input for each dataset via user interface (see example in Supplementary Figs. 1 and 2). We have used this function to optimise granularity choice for our datasets in order to enhance k-NN classification by setting a > 1 cluster and > 5 similar nodes per cluster criteria.

### Statistical analysis

Prism 9 (GraphPad, USA) was used to analyse and graph the data. Data was analysed using paired t-test and two-way ANOVA with post-hoc analysis as described in the relevant figure legends. Survival curves were generated and compared using the Log-rank Mantel-Cox test. These survival curves were generated with view of comparing predictive value of different methodologies for image analysis, namely SUV versus network methods, therefore were not controlled for potential confounding variables (e.g. sex or treatment methods) as those were present in both analysis methodologies. Furthermore, the multivariate Cox proportional hazards regression analysis model was used to evaluate the effects of key confounders (sex, age and chemotherapy treatment) on SUV and network analysis outcomes. Graphs are presented as mean ± SEM and differences are considered statistically significant at *p* < 0.05.

## Results

**Fig. 1:**
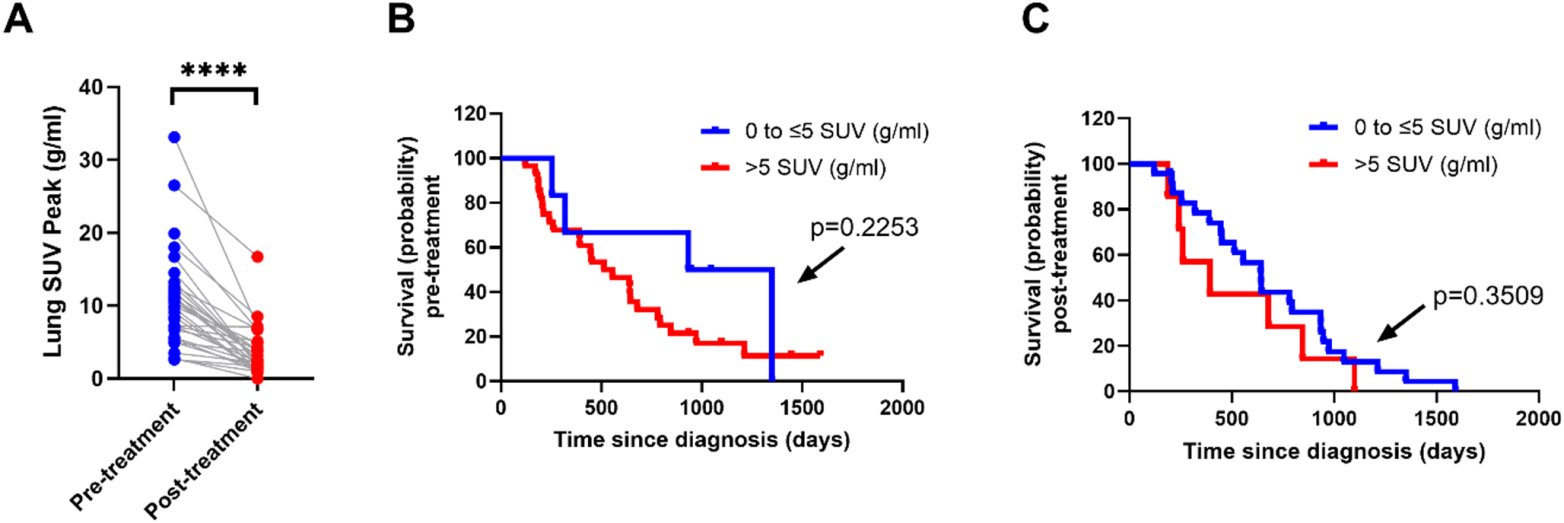
Conventional [^18^F]FDG PET SUV analysis of NSCLC patients. **A** Lung SUV peak values before and after chemoradiotherapy show a significant reduction in [^18^F]FDG uptake at the primary tumour site. Paired t-test with significance threshold set at *p* < 0.05 was used to analyse data in panel (A). *p = 0.05, **p = 0.01, ***p = 0.001, and ****p = 0.0001. Survival curves calculated based on lung SUV peak PET results before (**B**) and after (**C**) chemoradiotherapy show non-significant differences between ≤ 5 g/mL SUV peak and > 5 g/mL SUV peak groups. Log-rank Mantel-Cox test used to analyse panels (**B**) and (**C**) with significance threshold set at *p* < 0.05

Analysis of the routine clinical SUV measurements showed that lung SUV peak significantly decreases after chemoradiotherapy (Fig. 1A), demonstrating the effect of treatment in reducing glucose uptake at the primary tumour site. Neither lung SUV peak of the primary tumour measured at pre-treatment (Fig. 1B) or post-treatment (Fig. 1C) [^18^F]FDG PET scan provided significant prognostic value on patient survival when using the SUV cut-off of 5, previously identified as a significant SUV threshold when investigating patient survival in an all-patient analysis (stage II to IV) included in the ACRIN 6668 study [11].

**Fig. 2:**
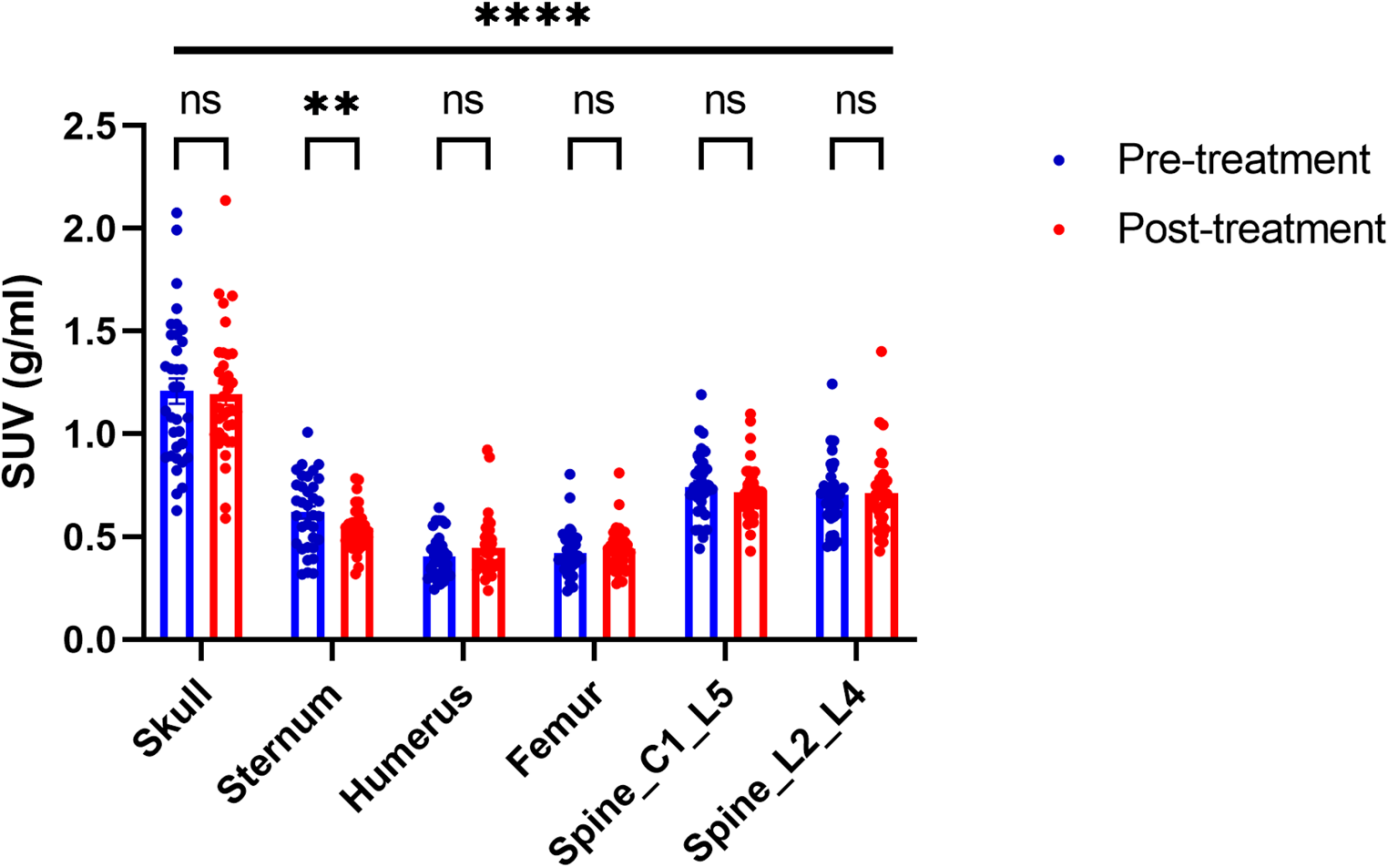
[^18^F]FDG uptake measured in different bones of stage IIIB NSCLC patients. Significant differences were measured in the sternum before and after chemoradiotherapy. Furthermore [^18^F]FDG uptake was significantly different for all bones at both the pre- and post-treatment analysis (indicated by the significance bar above all bones). A two-way ANOVA with Šídák’s multiple comparisons test was conducted, using a significance threshold set at *p* < 0.05. Data presented as individual scatter plots overlaid onto a bar graph with mean ± SEM error bar (*n* = 34 patients)

The SUV results differed significantly between different bones, for both pre- and post-treatment PET analysis, with a particularly high skull SUV (Fig. 2). Both spinal regions showed similar SUV results. The humerus and the femur also showed similar SUV to each other, though different to the spinal regions. There were no significant differences in SUV of individual bones between the pre-treatment and post-treatment values in lung cancer patients, except for a significant decrease in sternum SUV after treatment (Fig. 2).

**Fig. 3:**
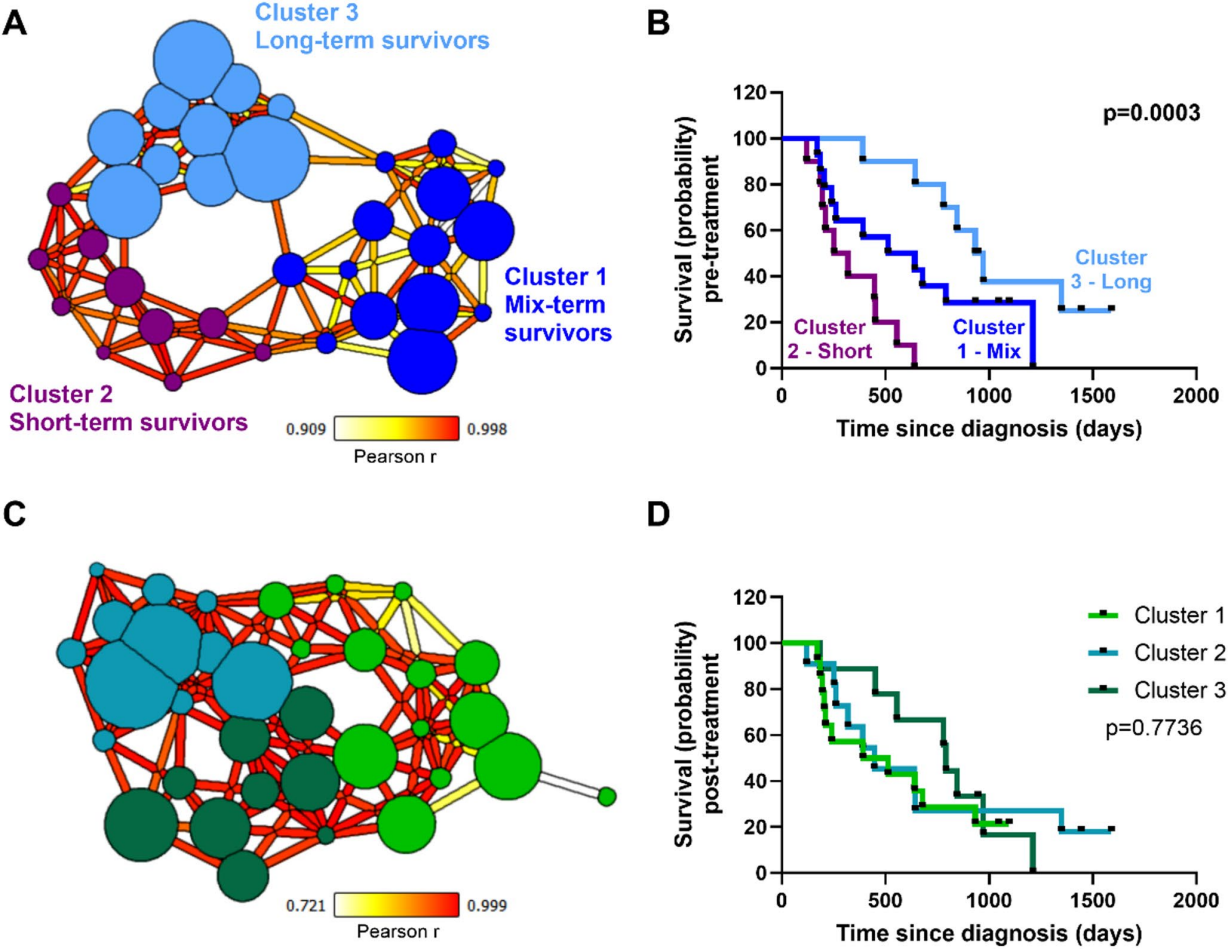
Network analysis of [^18^F]FDG PET skeletal system glucose metabolism in NSCLC patients. **A** Pre-treatment [^18^F]FDG PET skeletal network shows three distinct clusters: purple cluster containing predominately short-term NSCLC survivors, light blue cluster containing predominately long-term NSCLC survivors, and dark blue cluster containing a mixture of survivors (from short to long term). Network node size indicates survival period post-diagnosis. **B** Survival curves calculated based on PET skeletal metabolism data collected before chemoradiotherapy show significant differences between skeletal metabolism clusters identified in (A). **C** Post-treatment [^18^F]FDG PET skeletal network shows three distinct clusters, but all containing a mixture of survivors (from short to long term). Network node size indicates survival period post-diagnosis. **D** Survival curves calculated based on PET skeletal metabolism data collected after chemoradiotherapy show no significant differences between skeletal metabolism clusters identified in (C). Log-rank Mantel-Cox test used to analyse panels (B) and (D) with significance threshold set at *p* < 0.05

 Network analysis revealed subject-specific skeletal glucose metabolism in lung cancer patients with prognostication capability (Fig. 3) that was not observed using conventional SUV analysis of the same patient cohort (Fig. 2). [^18^F]FDG PET pre-treatment network shows three, moderately tight clusters, suggesting patients are closely correlated within clusters, but not between clusters (Fig. 3A). These newly identified clusters are significant predictors of patient survival (Fig. 3B). This was also observed when conducting Cox regression to adjust for key confounders (Supplementary Tables 1 and 2, Supplementary Fig. 3). Cox analysis also revealed sex was a significant factor in predicting patient survival from pre-treatment network analysis. The post-treatment network, however, shows the opposite effect. There are also three clusters, but these share many edges between them, showing that similarities within clusters and differences between clusters are not as pronounced as observed in the pre-treatment PET network (Fig. 3C). Contrary to the pre-treatment clusters, the post-treatment clusters identified by network analysis are not significant predictors of patient survival (Fig. 3D). This was also observed when conducting Cox regression to adjust for key confounders (Supplementary Tables 3 and 4, Supplementary Fig. 4). Importantly, there is a homogenisation of patients’ survival intervals in the post-treatment network (Fig. 3C) versus the pre-treatment network (Fig. 3A), suggesting systemic skeletal shift in glucose metabolism following chemoradiotherapy.

**Fig. 4:**
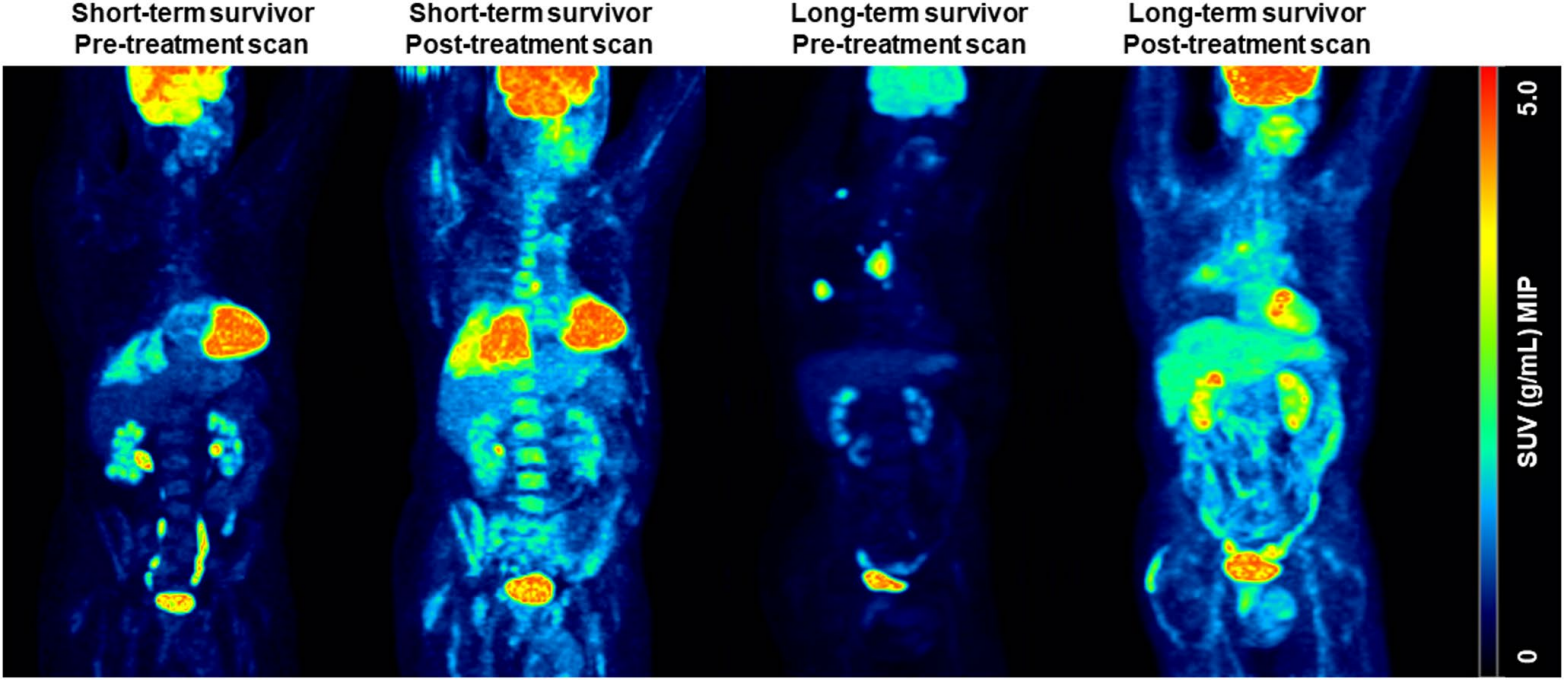
Representative maximum intensity projection (MIP) standardised uptake value (SUV) [^18^F]FDG PET images of NSCLC patients before and after treatment. From left to right: Short-term survivor [^18^F]FDG PET image pre-treatment; Short-term survivor [^18^F]FDG PET image post-treatment; Long-term survivor [^18^F]FDG PET image pre-treatment; and Long-term survivor [^18^F]FDG PET image post-treatment. Note significantly visible bone uptake in the pre- and post-treatment [^18^F]FDG PET image of the short-term survivor versus long-term survivor

Guided by the network analysis results, the assessment of whole-body [^18^F]FDG PET images showed a more pronounced bone radiotracer uptake in pre- and post-treatment short-term survivor scans compared with pre- and post-treatment long-term survivor scans (Fig. 4). This agrees with lung tumour response to chemoradiotherapy, but it is distinct from other multi-organ and multi-system changes before and after chemoradiotherapy. For example, liver and brain changes in [^18^F]FDG before and after chemoradiotherapy follow similar trends towards increase in short- and long-term survivors, albeit with different severities.

## Discussion

This study aimed to explore the utility of network analysis of bone glucose metabolism in the prognostication of lung cancer patients. Collected data showed that skeletal energy networks can serve as prognostic markers for NSCLC patients, while commonly used clinical measurements have limited ability to predict patient survival, despite providing useful metrics to assess treatment effects at the primary tumour site.

The significantly lower post-treatment lung SUV peak shows the high efficacy of chemoradiotherapy in reducing general tumour glucose uptake by damaging cell replication mechanisms [35]. However, lung SUV peak values at pre- and post-treatment do not seem to predict life expectancy. Previous studies have proposed the use of lung SUV peak at the post-treatment scan as prognostic marker of lung cancer, but recognised they could not identify a clinically significant cut-off value for post-treatment SUV other than a high post-treatment SUV portends a poor outcome [11]. Furthermore, the impact of post-treatment [^18^F]FDG PET in therapeutic decision making in the clinical setting is limited, as ideally patient allocation to treatment group would be personalised pre-treatment start.

Although higher glucose uptake is linked to a greater malignancy and hence, a worse survival outcome, reviews conflict on whether tumour SUV is an accurate predictor for disease development [11, 36]. This could be due to the metabolic reprogramming of cancer cells or dormant cancer cells located elsewhere in the body, which could cause a recurrence down the line [37, 38]. A single measurement at the site of the tumour may not be a very accurate representation of the disease. Cancer reprogramming affects whole-body glucose metabolism [12] so considering its effect on other important metabolic sites, such as the skeleton, may provide better insight into disease progression. Glucose metabolism has been shown to be altered in several organ systems in patients undergoing chemotherapy, including the brain [39], the heart [13, 40], the liver [41], and the spleen [42]. In fact, recent research conducted using the ACRIN6668 data shows how cardiac SUV can be a predictor of overall survival in NSCLC patients [13]. Thus, more insight into the effects of cancer at a systems level may provide a useful therapeutic tool in assessing disease progression in patients. This study focused on the skeletal system in particular for its role in the immune system and whole-body glucose regulation.

Conventional PET SUV analysis reveals differences in the glucose metabolism of individual bones, supported by previous research [23]. It is particularly higher in the skull potentially due to its proximity to the brain. The higher SUV uptake between spinal regions versus the humerus and femur could be due to their different composition. [^18^F]FDG SUV tends to be higher in trabecular bone compared to cortical bone, due to its increased surface area and higher number of osteoblasts, making it a more metabolically active site [16]. Vertebrae are made up of a ratio of 25:75 cortical to trabecular bone, while the femur is 50:50 cortical to trabecular bone [43].

Treatment seems to have no overall effect on individual bones SUVs, except for the sternum. Recent preclinical research showed that traumatic injury to an organ, such as a stroke in the brain, has effects on the surrounding bone and vice-versa. Confocal microscopy in murine stroke models shows direct interaction between the brain and the skull [44]. This also occurs between the heart, after suffering from myocardial infarction, and the sternum [45]. Furthermore, in a mouse model of lung cancer, data showed that osteoblasts remotely supply lung tumours with cancer-promoting neutrophils [22]. Thus, the changes in the sternum SUV may be attributable to a locally regulated interaction between the sternum and the diseased lungs, although follow-up mechanistic research targeting this hypothesis leveraged from this [^18^F]FDG human PET study is required.

Network analysis of [^18^F]FDG whole-body PET data revealed clear differences in the bone metabolic profiles of patients before and after treatment that could not be appreciated through conventional analysis. Treatment seems to have a homogenising effect on the PET bone networks, suggesting chemoradiotherapy negates the predisposition of certain patients for better or worse disease outcomes. Research on deletions on 9p21 has shown that certain gene deletions are associated with worse disease outcomes in NSCLC patients treated with immunotherapy, but do not affect disease outcomes in patients treated with chemoradiotherapy [46], highlighting the systemic and severe effects of chemoradiotherapy in cancer patients. Of greater significance are our data showing that having a particular initial bone metabolic profile can be predictive of longer survival. This can be a powerful prognostic tool for NSCLC patients and has the potential to aid personalised allocation of patients into different treatment plans. Additionally, the loss of prognostication potential of bone networks post-treatment might relate to changes in the adaptive immunity rather than innate immunity of bones. This is because osteoclasts are germline encoded cells with innate immune roles [20]. In inflammation-induced bone resorption, cytokines such as RANKL, TNF-α, and M-CSF drive osteoclast differentiation and activation, a process known as osteoclastogenesis. Conversely, regulatory cytokines like IL-4, IL-10, and interferons help limit excessive bone loss, highlighting the dual role of cytokines in both promoting and restraining bone resorption. This dynamic balance underscores their potential as targets for maintaining bone homeostasis and developing immunotherapies [47]. Furthermore, osteoclasts themselves can secrete pro-inflammatory cytokines such as interleukin-8 (IL-8), which serve multiple roles in the bone-immune axis. IL-8 acts in an autocrine fashion to enhance osteoclast differentiation and activity, thereby amplifying bone resorption. This bidirectional communication between osteoclasts and immune cells not only sustains local inflammatory responses but also links systemic immune activation to skeletal remodeling. Simultaneously, IL-8 functions as a potent chemoattractant for neutrophils and other immune cells, facilitating their recruitment to the bone microenvironment [48]. Chemokines such as CCL3 and CCL9, secreted by osteoclasts, play critical roles in promoting osteoclast differentiation and migration. Notably, CCL3 enhances osteoclastogenesis by signalling through CCR1 and CCR5 receptors and has been implicated in the osteolytic activity observed in pathological conditions such as multiple myeloma [49].

Interestingly, the distribution of the survival curves for pre-treatment [^18^F]FDG bone networks supports the role of heterozygotic or homozygotic genetic mutations affecting bone metabolism. For example, high-penetrance gene (HPG) pathogenic mutations, found in around a third of NSCLC patients, lead to more aggressive cancer. Patients with HPG mutations are more likely to experience cancer recurrence and have worse overall survival [50]. It is also known that chemoradiation can severely disrupt bone homeostasis [51] both by activation of immune and inflammatory responses as well as in response to systemic glucose changes [52]. These mechanisms likely underly the observed changes in bone metabolism before and after treatment reported here.

**Fig. 5:**
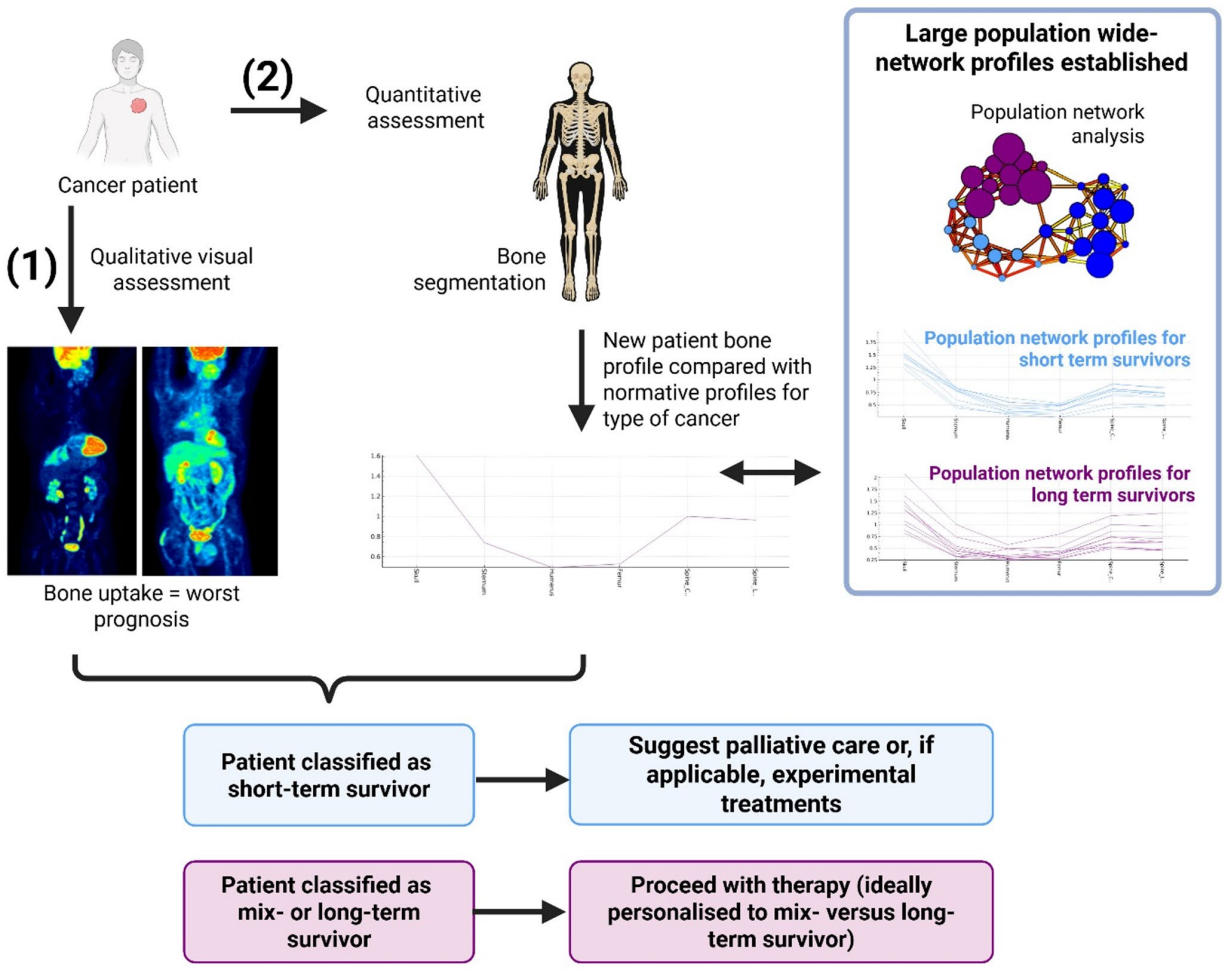
Envisioned step-by-step framework for clinical implementation of network analysis of whole-body PET data. Two possible routes: (1) a qualitative assessment based on bone uptake visible in the whole-body PET pre-treatment scan; or (2) a preferable, quantitative assessment based on network analysis. For route 2, the first step involves segmentation of patient’s bones. Then, bone SUV profiles are generated for the new patient and compared with different cluster SUV profiles obtained through large multi-centre clinical trials (the next step following this proof-of-concept report). Based on patient classification as short-term survivor (based on higher bone uptake on pre-treatment PET scan or network analysis), palliative care or, when applicable, experimental treatments could be proposed. Conversely, if the patient is classified as mix- or long-term survivor, the recommendation would be to proceed with therapy and preferably this would be personalised for mix- versus long-term survivors. Future work with large clinical samples would allow for prioritisation of a given treatment regimen over another for mix- and long-term survivors

Network analysis presents a promising new approach to stratify patients based on PET bone glucose biomarkers, offering the potential to predict patient survival more accurately and early in the journey of patient care (Fig. 5). By integrating network analysis with total- or whole-body PET, this method could provide a comprehensive overview of metabolic activity patterns across multiple tissue sites, facilitating a more nuanced understanding of systemic and multi-organ disease progression. Once the different clusters of patients are mapped out using a normative framework, this approach could aid in identifying patient subgroups that could benefit from targeted therapies, ultimately supporting more personalised treatment decisions (Fig. 5). These findings highlight the potential of network analysis as an additional tool in clinical practice, enabling tailored treatment strategies that improve patient outcomes. The use of network analysis is relatively new in the field of whole-body PET image analysis, however, other studies using alternative network analysis methodologies have also identified important metabolic dysfunction from a systemic perspective in lung cancer patients [12]. Therefore, corroborating the importance of network analysis in disease management.

Despite the important results reported in this proof-of-concept study, future studies with a larger sample size would be needed to improve the generalizability of the results. Due to the nature of the network analyses, standard power calculations to help determine the sample size needed for a larger study are challenging. However, previous reports using network analysis approaches in different biomedical applications suggest the method can be robust and stable for different sample sizes between 15 and 50 [53]. Recently, we have also tested the sensitivity of bone network analysis to different conditions and found these were robust to changes in data from different PET measurements (single subject versus multiple subjects), increased noise, and shortened scan length [33]. Our clustering strategy could also be improved, for example, by dynamic multi-clustering granularity optimisation, as previously reported [34]. However, our iterative optimisation of single granularity has provided good proof-of-concept evidence of the value of network analysis in PET/CT clinical imaging, warranting further larger investigations in the future. Interestingly, power calculations for the conventional SUV for pre- versus post-treatment analysis suggest *n* numbers between 79 (for the sternum analysis only) and 8727 (for the skull analysis) would be recommended. Notwithstanding, further work with larger sample sizes and different lung cancer patient cohorts is required to fully validate our proof-of-concept findings.

A limitation of this study is that not all patients received the same treatment regimen, which may introduce variability in results. However, this reflects the reality of clinical practice, where treatment regimens are often tailored to individual patient characteristics, comorbidities, and physician preferences. This study acknowledges that there are several potential confounding factors, including metabolic disorders, prior treatments, and underlying conditions such as inflammation, which may influence skeletal PET results. Whilst post-treatment metabolic changes and their potential association with innate immunity are proposed, further validation is required, and the link between these shifts and innate immune mechanisms remains hypothetical and requires further research.

Another limitation of the study is its retrospective nature. Using an open-source database usually entails some missing data, which may lead to patient exclusion or missing data points in certain analyses. The nature of the study also means no access to more biological and lifestyle factors, such as genetic information, smoking habits or performance status and acquiring this data now is no longer possible. These factors are known to influence survival outcomes, and their absence limits the ability to adjust for key confounding variables. Therefore, the next step in this line of research would be to conduct a larger prospective study. This would help overcome some of the previously stated limitations.

## Conclusion

Overall, this study presents a novel method of quantifying changes in aberrant glucose metabolism in lung cancer patients from a systems-level perspective by making use of network analysis as opposed to conventional statistical analysis. It reveals the potential use of pre-treatment bone metabolic networks as clinical tools to predict prognosis and recommend personalised treatments for lung cancer patients. However, results are correlational; and further validation in larger, prospective cohorts is required to fully establish the prognostic potential of skeletal metabolic networks. Further research, including genomic and immune profiling may help elucidate the biological mechanisms between inter-patient differences.

## Electronic supplementary material

Below is the link to the electronic supplementary material.

Supplementary Material 1

## Data Availability

The datasets analysed during the current study are available in the TCIA repository (https://www.cancerimagingarchive.net/collection/acrin-nsclc-fdg-pet/).
