# Supplementary material for: Innate [18F]Fluorodeoxyglucose PET bone networks of lung cancer patients predict survival
Source: Eur J Nucl Med Mol Imaging. 2025 Jun 5;52(13):4952–62. doi: 10.1007/s00259-025-07388-8 (PMC12589343; doi:10.1007/s00259-025-07388-8)
Supplement: Supplementary file 1 — Supplementary Material 1 [file 259_2025_7388_MOESM1_ESM.docx]

**Supplementary File**

**Innate [^18^F]Fluorodeoxyglucose PET bone networks of lung cancer patients predict survival**

Rucha Ronghe^1†^, Teresa Crespo^2†^, Catriona Wimberley^3,4,5^, Karla Suchacki^3,6^, and

Adriana A. S. Tavares^3,4^*

**^†^These authors contributed equally to this work.**

^1^ Edinburgh Medical School, University of Edinburgh, 49 Little France Crescent, Edinburgh, EH16 4SB, UK

^2^ Edinburgh Medical School: Biomedical Sciences, University of Edinburgh, Old Medical School (Doorway 3), Teviot Place, Edinburgh, EH8 9AG, UK

^3^ British Heart Foundation-University of Edinburgh Centre for Cardiovascular Science, University of Edinburgh, 47 Little France Crescent, EH16 4TJ, Edinburgh, UK

^4^ Edinburgh Imaging, University of Edinburgh, EH16 4TJ, Edinburgh, UK

^5^ School of Physics and Astronomy, University of Edinburgh, EH9 3JW, Edinburgh, UK

^6^ SRUC, The Roslin Institute, Edinburgh, UK.

***Corresponding author:**

Adriana A.S. Tavares

Centre for Cardiovascular Science, Queen's Medical Research Institute, 47 Little France Crescent, Edinburgh EH16 4TJ, UK

[adriana.tavares@ed.ac.uk](mailto:adriana.tavares@ed.ac.uk)


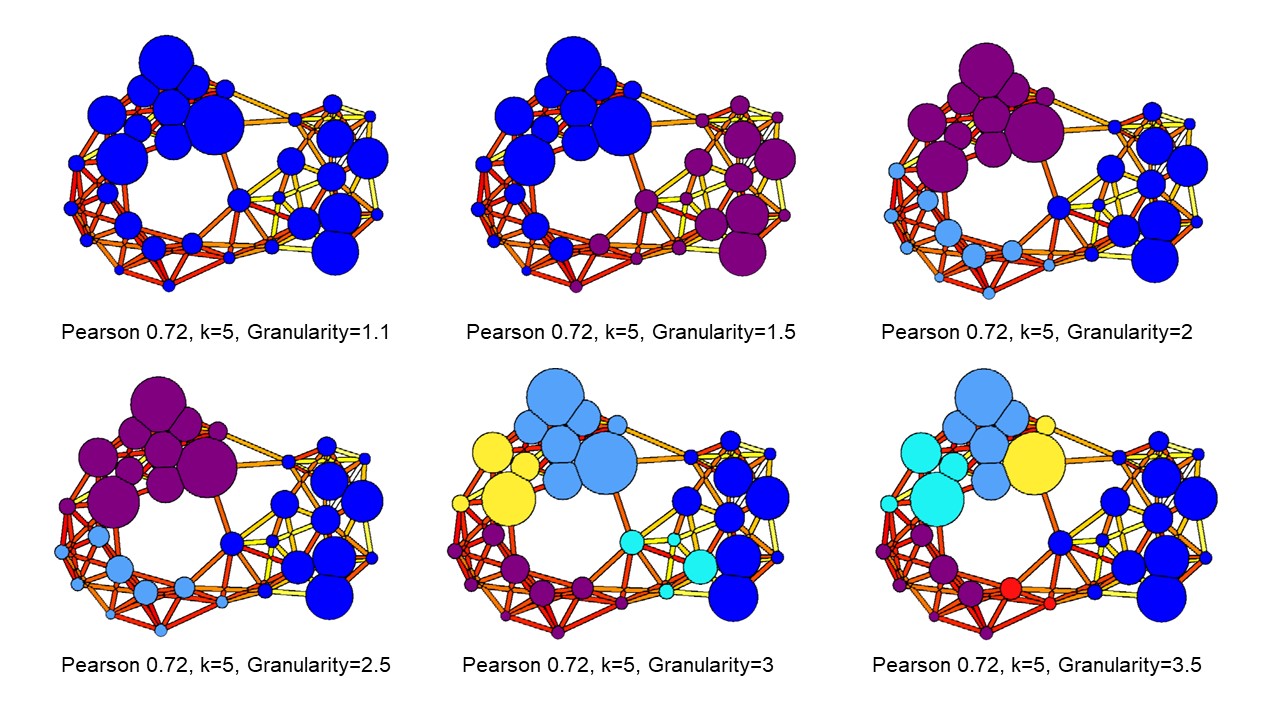


**Supplementary Figure 1.** Optimisation of single granularity parameter for networks derived from pre-treatment PET datasets.


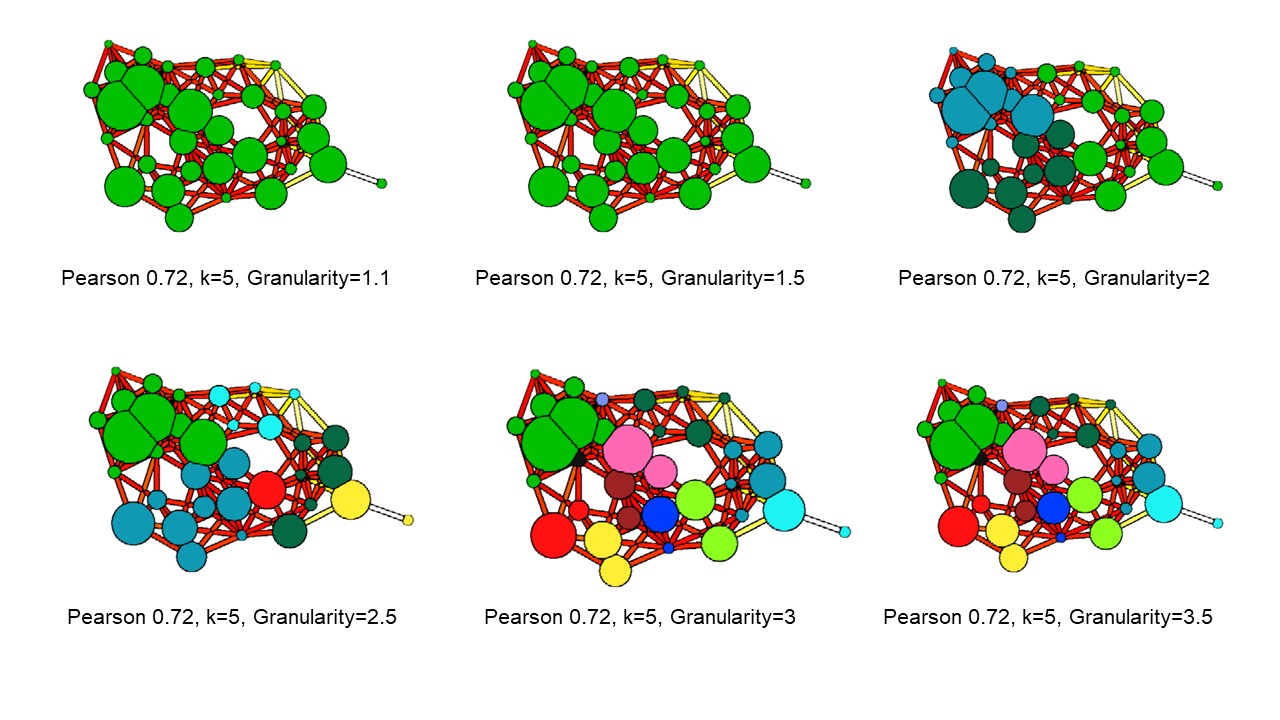


**Supplementary Figure 2.** Optimisation of single granularity parameter for networks derived from post-treatment PET datasets.

**Supplementary Table 1.** Multi-variate Cox analysis of lung SUV results from pre-treatment PET scans. Legend: CI=confidence interval.

| **Variable** | **Hazard ratios** | **95% CI** | **p value** |
| --- | --- | --- | --- |
| **Age** | 1.02 | 0.96 to 1.08 | 0.60 |
| **Sex (male reference)** | 0.48 | 0.17 to 1.30 | 0.16 |
| **SUV (<5 reference)** | 0.38 | 0.10 to 1.18 | 0.12 |
| **Chemotherapy (Cisplatin+Etoposide reference)** | | | |
| Carboplatin+Paclitaxel | 0.84 | 0.24 to 3.06 | 0.78 |
| Carboplatin+Taxotere | 1.10 | 0.24 to 4.63 | 0.90 |
| Cisplatin+Irinotecan | 0.48 | 0.10 to 1.91 | 0.31 |
| Cisplatin+Navelbine | 2.66 | 0.25 to 23.63 | 0.38 |
| Carboplatin+Navelbine | 2.63 | 0.12 to 24.37 | 0.44 |
| Cisplatin+Paclitaxel | 0.52 | 0.03 to 3.21 | 0.55 |
| Carboplatin+Etoposide | 3.63 | 0.18 to 26.42 | 0.26 |
| Navelbine | 2.15 | 0.07 to 38.35 | 0.61 |

**Supplementary Table 2.** Multi-variate Cox analysis of bone network results from pre-treatment PET scans. Legend: CI=confidence interval. *p<0.05, ***p<0.001.

| **Variable** | **Hazard ratios** | **95% CI** | **p value** |
| --- | --- | --- | --- |
| **Age** | 0.98 | 0.92 to 1.04 | 0.56 |
| **Sex (male reference)** | 0.29 | 0.09 to 0.87 | 0.03* |
| **Cluster (reference to long-term survivors)** | | | |
| Short-term survivors cluster | 24.61 | 4.80 to 144.50 | 0.0002*** |
| Mix-term survivors cluster | 6.72 | 1.51 to 34.29 | 0.02* |
| **Chemotherapy (Cisplatin+Etoposide reference)** | | | |
| Carboplatin+Paclitaxel | 0.58 | 0.14 to 2.51 | 0.74 |
| Carboplatin+Taxotere | 0.36 | 0.05 to 2.40 | 1.06 |
| Cisplatin+Irinotecan | 0.15 | 0.02 to 1.12 | 1.80 |
| Cisplatin+Navelbine | 1.05 | 0.10 to 9.21 | 0.05 |
| Carboplatin+Navelbine | 0.62 | 0.03 to 6.27 | 0.37 |
| Cisplatin+Paclitaxel | 0.14 | 0.005 to 1.59 | 1.45 |
| Carboplatin+Etoposide | 0.42 | 0.02 to 4.36 | 0.67 |
| Navelbine | 3.78 | 0.09 to 127.20 | 0.75 |

**Supplementary Table 3.** Multi-variate Cox analysis of lung SUV results from post-treatment PET scans. Legend: CI=confidence interval.

| **Variable** | **Hazard ratios** | **95% CI** | **p value** |
| --- | --- | --- | --- |
| **Age** | 0.99 | 0.93 to 1.06 | 0.89 |
| **Sex (male reference)** | 0.63 | 0.19 to 1.97 | 0.43 |
| **SUV (<5 reference)** | 1.72 | 0.42 to 7.20 | 0.44 |
| **Chemotherapy (Cisplatin+Etoposide reference)** | | | |
| Carboplatin+Paclitaxel | 1.43 | 0.40 to 5.89 | 0.59 |
| Carboplatin+Taxotere | 1.45 | 0.26 to 7.45 | 0.65 |
| Cisplatin+Irinotecan | 0.53 | 0.09 to 2.54 | 0.44 |
| Cisplatin+Navelbine | 4.77 | 0.35 to 54.15 | 0.21 |
| Carboplatin+Navelbine | 4.84 | 0.21 to 50.77 | 0.22 |
| Cisplatin+Paclitaxel | 1.03 | 0.05 to 7.65 | 0.98 |
| Carboplatin+Etoposide | 9.51 | 0.42 to 96.27 | 0.07 |
| Navelbine | 3.51 | 0.07 to 116.00 | 0.49 |

**Supplementary Table 4.** Multi-variate Cox analysis of bone network results from post-treatment PET scans. Legend: CI=confidence interval.

| **Variable** | **Hazard ratios** | **95% CI** | **p value** |
| --- | --- | --- | --- |
| **Age** | 0.99 | 0.94 to 1.05 | 0.82 |
| **Sex (male reference)** | 0.48 | 0.17 to 1.28 | 0.15 |
| **Cluster (reference to Mixed Cluster 2)** | | | |
| Mixed Cluster 1 | 2.12 | 0.64 to 7.27 | 0.22 |
| Mixed Cluster 2 | 0.87 | 0.26 to 2.88 | 0.82 |
| **Chemotherapy (Cisplatin+Etoposide reference)** | | | |
| Carboplatin+Paclitaxel | 0.70 | 0.20 to 2.60 | 0.58 |
| Carboplatin+Taxotere | 1.10 | 0.23 to 4.80 | 0.90 |
| Cisplatin+Irinotecan | 0.27 | 0.04 to 1.40 | 0.13 |
| Cisplatin+Navelbine | 4.97 | 0.46 to 46.17 | 0.16 |
| Carboplatin+Navelbine | 3.76 | 0.17 to 37.00 | 0.29 |
| Cisplatin+Paclitaxel | 0.75 | 0.04 to 5.11 | 0.80 |
| Carboplatin+Etoposide | 2.04 | 0.09 to 18.54 | 0.57 |
| Navelbine | 2.28 | 0.07 to 44.84 | 0.60 |


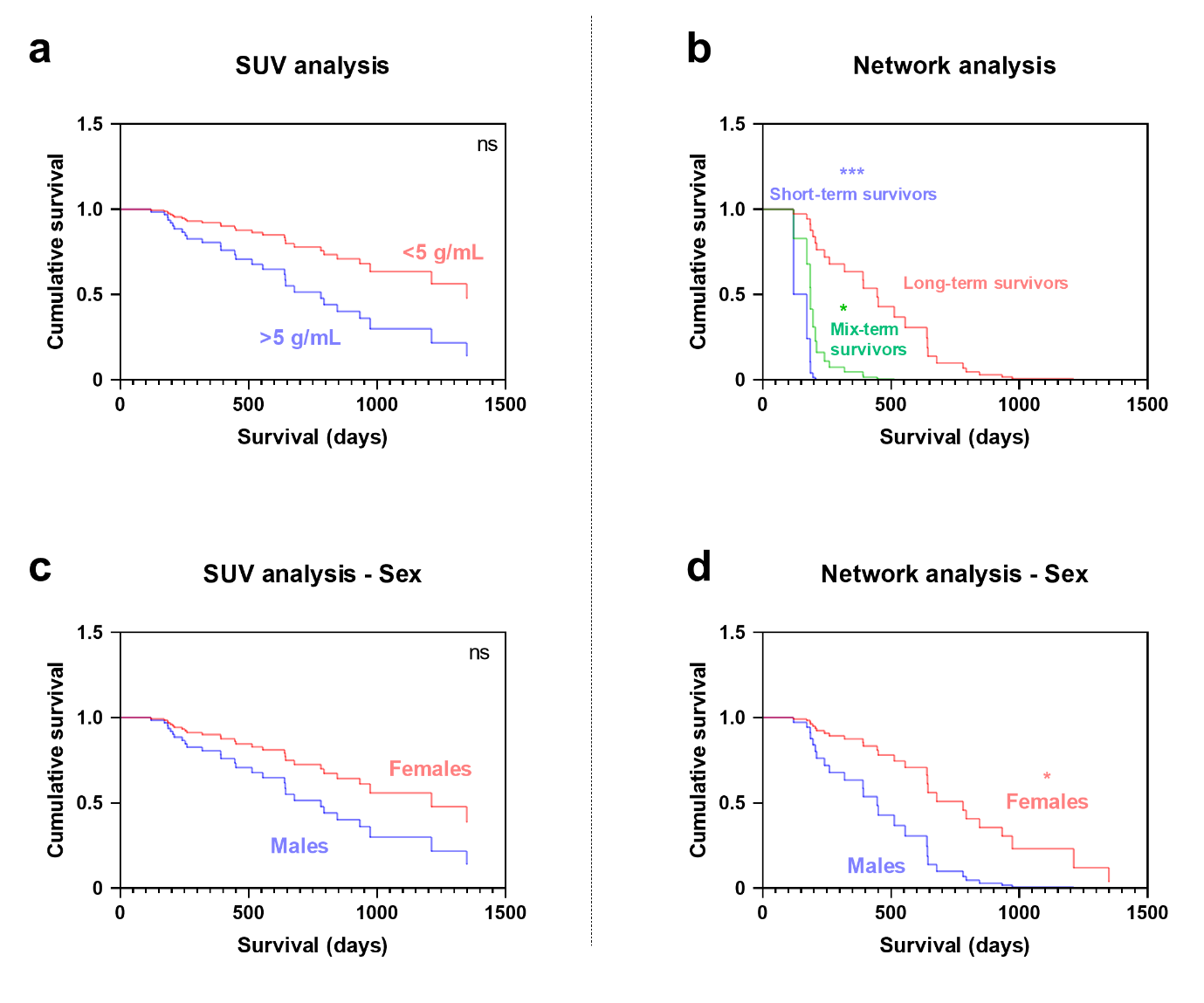


**Supplementary Figure 3.** Multi-variate Cox analysis for pre-treatment SUV results using threshold of 5 g/mL (a), network clusters (b), sex-adjusted SUV results (c), and sex-adjusted network analysis results (d). Cox regression models were censored as dead/alive with time variable being survival in days. Significant differences between cumulative survival analysis for different variables are presented as *p<0.05 and ***p<0.001. ns=non-significant.


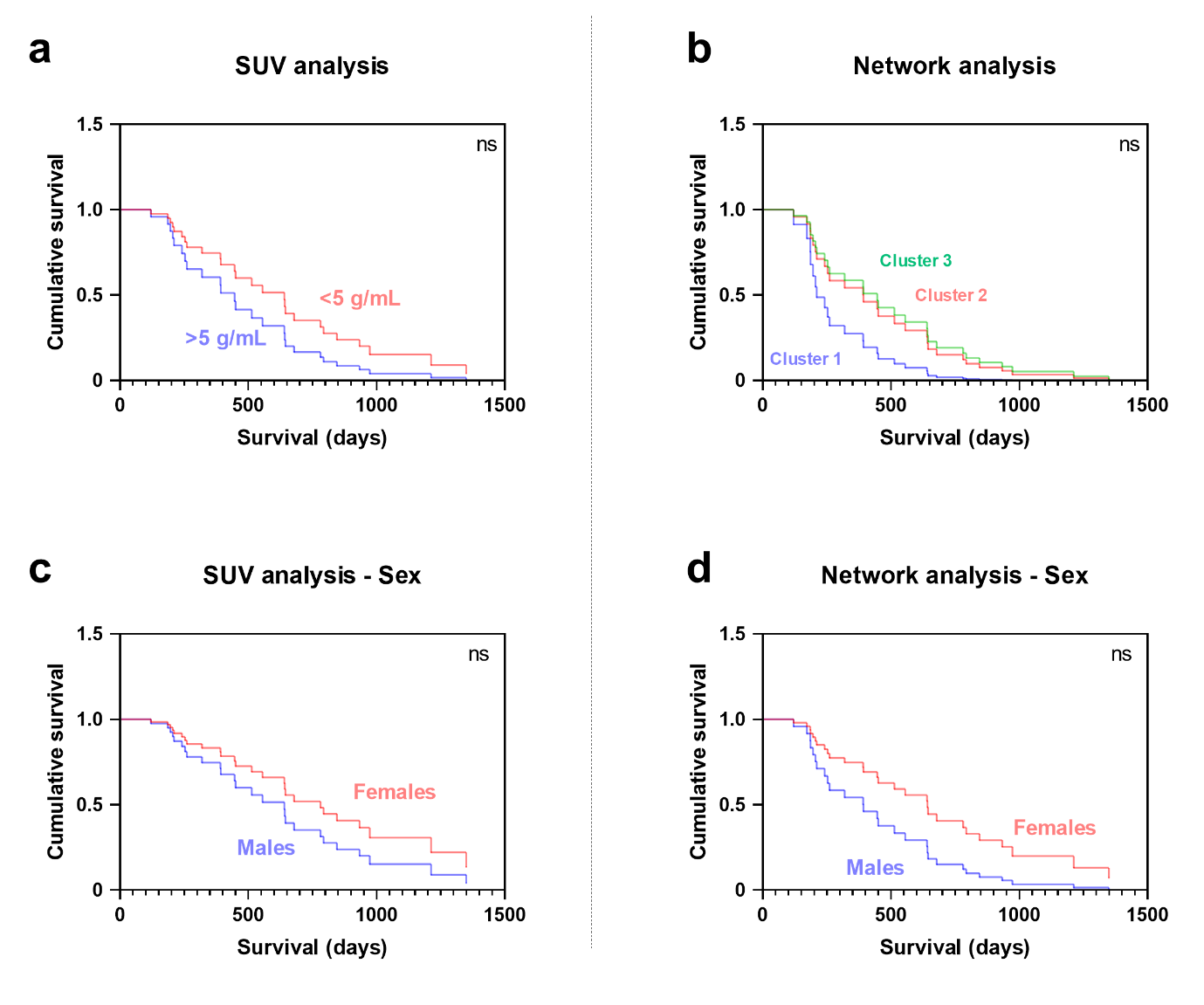


**Supplementary Figure 4.** Multi-variate Cox analysis for post-treatment SUV results using threshold of 5 g/mL (a), network clusters (b), sex-adjusted SUV results (c), and sex-adjusted network analysis results (d). Cox regression models were censored as dead/alive with time variable being survival in days. Significant differences between cumulative survival analysis for different variables are presented as *p<0.05 and ***p<0.001. ns=non-significant.
